# Differences in Maternal and Neonatal Outcomes Between Pregnancies Following Intrauterine Insemination (IUI) and In Vitro Fertilization (IVF): A Review

**DOI:** 10.3390/jcm14248889

**Published:** 2025-12-16

**Authors:** Panagiotis Cherouveim, Esra Cetin, Shagun Tuli, Taylor Lombard, Youssef Youssef, Gaby Moawad, Jean Marc Ayoubi, Anis Feki

**Affiliations:** 1 Department of Obstetrics and Gynecology, Hurley Medical Center, Michigan State University, Flint, MI 48503, USA; 2 College of Human Medicine, Michigan State University, Flint, MI 48503, USA; 3 Division of Minimally Invasive Gynecologic Surgery, Department of Obstetrics and Gynecology, Maimonides Medical Center, Brooklyn, NY 11219, USA; 4 Department of Obstetrics and Gynecology, The George Washington University School of Medicine & Health Sciences, Washington, DC 20037, USA; 5 Department of Gynecology and Obstetrics and Reproductive Medicine, Hospital Foch, 92150 Paris, France; 6 Department of Obstetrics and Gynecology, Fribourg University Hospital, 1708 Fribourg, Switzerland

**Keywords:** in vitro fertilization, intrauterine insemination, maternal outcomes, neonatal outcomes

## Abstract

Pregnancies following Assisted Reproductive Technology (ART) are associated with increased risk for adverse maternal and neonatal outcomes. However, it is not clear if these associations are derived from infertility or fertility treatment itself. The goal of this review is to investigate if there are differences in maternal and neonatal outcomes between pregnancies conceived through either intrauterine insemination (IUI) or in vitro fertilization (IVF) to obtain an estimate of the effect of the fertility treatment. We retrospectively reviewed the literature for original studies comparing maternal and neonatal outcomes in pregnancies following IUI to those following IVF. To reduce confounding, our synthesis focuses on singleton gestations, while excluding donor sperm and multifetal pregnancies. Findings are interpreted alongside recent ART vs. non-ART evidence to contextualize potential treatment-related effects. Most studies suggest no difference in maternal outcomes, but outcomes were inconsistently reported between studies. Neonatal outcomes were more consistently reported, and there was less variation between results. Current evidence does not suggest a large difference in neonatal outcomes between IVF and IUI. However, almost all studies have significant limitations that hinder clear conclusions. To summarize, even though the current literature does not suggest significant differences in maternal and neonatal outcomes following IVF and IUI, further high-quality studies are needed to establish a definite correlation.

## 1. Introduction

Assisted Reproductive Technology (ART) is a safe and effective solution for women pursuing parenthood. However, when compared to spontaneously conceived pregnancies, pregnancies following ART have an increased risk for obstetric and neonatal complications [1], birth defects [2], and epigenetic disorders [3]. Pathophysiological mechanisms responsible for these associations remain to be determined [3]. Maternal complications associated with ART include hypertensive disorders of pregnancy [4], gestational diabetes mellitus [5], placenta previa [6], placental abruption, antepartum hemorrhage, postpartum hemorrhage, polyhydramnios, oligohydramnios [5], and cesarean sections [7]. Additionally, adverse perinatal outcomes associated with ART are preterm and very preterm birth [8], low and very low birth weight [4], small for gestational age, perinatal mortality [5], and a variety of birth defects [2,9].

While these findings may vary within the literature, they were initially attributed to the presence of multiple gestation as a major influence [10,11]. Many of these associations, however, continue to be present even after controlling for multiple gestations [4]. Other possible contributing factors are the diagnosis of infertility, the effects of in vitro culture and laboratory techniques, and the stimulation protocols and associated treatment medications. In most studies, conclusions were reached by comparing infertile patients to fertile controls who conceived spontaneously, making it challenging to conclude whether these differences are related to the infertility diagnosis or the medical ART treatments.

There were two recent studies based on data from the state of Massachusetts [12,13]. The former [12] concluded that PTB and LBW were higher in the infertile patients compared to fertile, and even higher if ART was used, while the latter [13] found a correlation between infertility-related diagnoses and perinatal morbidities such as PTB and LBW. Nonetheless, these studies were trying to determine the effect of ART and did not take into account the different ovarian stimulation protocols or contained incomplete information on patient characteristics, such as infertility diagnosis. The two populations differ substantially, potentially confounding results and limiting the validity of the conclusions. Older age [1], infertility diagnoses, and co-existing medical risk factors may contribute to the observed differences in adverse maternal and neonatal outcomes. Additionally, the state of Massachusetts has established a special policy for ART coverage, which is not necessarily representative of the general population. Finally, the impact of the type of treatment and/or ovarian stimulation on these outcomes has not been fully evaluated in previously published studies.

These limitations could be addressed by comparing maternal and neonatal outcomes between pregnancies following different fertility treatments. Pregnancies within these groups will be more homogeneous with respect to infertility rates and severity. This review explores the potential differences in outcomes between pregnancies following in vitro fertilization (IVF) and intrauterine insemination (IUI). It is important to note that much of the comparative IVF–IUI literature was conducted 10–20 years ago, prior to widespread adoption of modern ART practices such as vitrification, single-blastocyst transfer, milder ovarian stimulation, and optimized laboratory culture systems. These substantial changes in technology and protocols limit the applicability of earlier study findings to contemporary practice. Therefore, the interpretation of historical data must be made cautiously, as the true effect of treatment modality may differ in current settings.

We included data from singleton-only comparisons for the primary conclusions, while excluding twin and donor-sperm pregnancies to minimize confounding since they have both been associated with adverse obstetric and perinatal outcomes [14,15]. To strengthen the relevance and contemporary framing of this review, we also situate IUI–IVF comparisons within the broader literature that contrasts ART with non-ART (spontaneously conceived) pregnancies. Multiple high-quality syntheses indicate that ART-conceived offspring experience elevated risks for prematurity, low birthweight, and certain longer-term health differences compared with non-ART conceptions, although disentangling treatment effects from underlying infertility remains challenging [16,17]. Referencing these data clarifies why direct IUI–IVF comparisons, while valuable, should be interpreted alongside non-ART comparators to estimate the incremental impact of treatment intensity and laboratory exposure.

## 2. Methods

A structured literature review was performed using PubMed, Embase, and Web of Science. Search terms included combinations of: ‘in vitro fertilization’, ‘IVF’, ‘intrauterine insemination’, ‘IUI’, ‘maternal outcomes’, ‘neonatal outcomes’, ‘perinatal outcomes’, and ‘assisted reproduction’. The search included publications from 1990 to 2025.

Inclusion criteria included: (1) original studies directly comparing maternal and/or neonatal outcomes in pregnancies conceived via IUI vs. IVF; (2) human studies; (3) studies reporting outcomes separately for singleton pregnancies when possible. Exclusion criteria included: studies limited to donor sperm cycles, multifetal gestations, or studies without separable IVF vs. IUI outcome data.

References from eligible studies were also screened for additional articles. Statistical synthesis of the literature review results was unable to be conducted, given the heterogeneity of the results reported in the literature. Statistical methods from each study were extracted as reported when available.

## 3. Processes and Mechanisms Distinguishing IVF vs. IUI

IVF introduces laboratory and endometrial-preparation exposures (controlled ovarian stimulation intensity, oocyte retrieval, ICSI, embryo culture duration and media, fresh vs. frozen transfer, luteal support, and endometrial programming) [18] that are largely absent or attenuated with IUI, where risk is dominated by ovulation induction (oral agents vs. gonadotropins), multifollicular development, and trigger timing [19]. These differences offer a biologic rationale for graded risks across spontaneous conceptions, IUI, and IVF, and motivate separating: (1) stimulation-related effects (e.g., follicle number, estradiol levels) [19], (2) laboratory exposure effects (e.g., extended culture) [20,21], and (3) endometrial milieu (e.g., programmed vs. natural cycles) [18,22,23,24]. Our synthesis therefore reports outcomes by treatment class while highlighting process-level modifiers when studies allowed (e.g., oral vs. injectable induction for IUI; fresh vs. frozen transfer for IVF) [24].

## 4. Maternal Outcomes

Table 1 lists study-level estimates on maternal outcomes, while interpretation is provided in the paragraphs below. A total of 6 studies reported maternal outcomes following IVF and IUI [25,26,27,28,29,30]. Main outcomes of interest include the mode of delivery [25,26,27,28], antepartum hemorrhage [25,28,30], and hypertensive disorders of pregnancy [25,26,27,28,29]. These outcomes were selected to be included as they were reported in at least 3 studies. Maternal outcomes that were reported and their differences between groups in various studies were inconsistent, making definitive conclusions challenging.

### 4.1. Mode of Delivery

In most studies, cesarean section rates do not seem to differ between IUI and IVF. However, Poon et al. [28] found cesarean section rates to be increased among IVF-conceived pregnancies compared to IUI, by indirectly comparing section rates after IVF and IUI to natural conception (NC) (Table 1).

### 4.2. Antepartum Hemorrhage

De Sutter et al. [25] found no significant differences in rates of antepartum hemorrhage between IVF and IUI, when stratified by trimester. Poon et al. [28] reported a higher risk for antepartum hemorrhage with IVF compared to IUI, by indirectly comparing IVF and IUI to NC. Wang et al. [30], also report higher antepartum hemorrhage rates with IVF-conceived pregnancies compared to those conceived with IUI treatments; however, this was a secondary outcome of the original study (Table 1).

### 4.3. Hypertensive Disorders of Pregnancy

Two studies support no difference in hypertensive disorders of pregnancy between IVF and IUI [25,27]. Nuojua-Huttunen et al. [26] do not report a *p*-value for the comparison; however, the rates for hypertensive disorders of pregnancy are low for both IVF and IUI (Table 1). Wessel et al. [29] also do not report a *p*-value for the IVF vs. IUI comparison. However, neither of the two groups differed significantly from NC in terms of rates of hypertensive disorders of pregnancy (*p* > 0.05). Poon et al. [28] report a higher risk for preeclampsia with IVF compared to IUI, by indirectly comparing IVF and IUI to NC (Table 1).

### 4.4. Other

Malchau et al. [27] additionally described an increased risk for placenta previa in IVF-conceived pregnancies compared to IUI (2.3% for IVF vs. 1.1% for IUI, *p* < 0.001). Nuojua-Huttunen et al. [26] reported risks for placenta previa and placenta abruption that were all < 1% (placenta previa: 0.7% for IVF vs. 0% for IUI; placenta abruption: 0.7% for IVF vs. 0% for IUI), and concluded that there were no significant differences, despite not reporting *p*-values.

Two additional maternal outcomes were reported in the literature—preterm contractions [25] and gestational diabetes [26,28]—with no significant difference between IVF- and IUI-conceived pregnancies.

### 4.5. Summary

In summary, the evidence appears mixed, and current data do not suggest a large difference in maternal outcomes. However, a clear conclusion for maternal outcomes in pregnancies following IVF and IUI could not be reached based on the current literature, as maternal outcomes were inconsistently reported in various studies.

## 5. Neonatal Outcomes

Table 2 contains a summary of the study-level estimates for neonatal outcomes. A total of 7 studies reported neonatal outcomes following IVF and IUI [25,26,27,28,29,30,31]. In a similar fashion to maternal outcomes, we included outcomes reported in at least 3 studies. Those included gestational age at the time of delivery [25,26,27,28,29,30,31], birth weight [25,26,27,28,29], neonatal intensive care unit (NICU) admission [25,26,27,28], APGAR scores [25,26,28], and perinatal mortality [26,27,28].

### 5.1. Gestational Age (GA) at Delivery

Seven studies [25,26,27,28,29,30,31] made comparisons of GA at delivery between groups. Three studies found no differences in GA either by directly comparing IVF and IUI [25], or by indirectly comparing IVF and IUI with NC [28,30]. Three more studies reported similar GAs at delivery for both IVF and IUI, but no *p*-values were reported for the comparison [26,29,31]. Malchau et al. [27] reported slightly lower GAs at delivery following IVF compared to IUI (Table 2).

### 5.2. Birth Weight (BW)

Five studies reported BW following IVF and IUI conceptions [25,26,27,28,29]. Two found no significant differences between IVF and IUI with either direct [25] or indirect comparisons [28]. Two more studies reported BWs at delivery for IVF and IUI, respectively, but without reporting *p*-values for the comparisons [26,29]. Malchau et al. [27] reported slightly lower BWs for IVF conceptions compared to IUI (Table 2).

### 5.3. NICU Admission

Four studies made comparisons of NICU admissions between groups [25,26,27,28]. Two found no significant differences between IVF and IUI [25,27]. Nuojua-Huttunen et al. [26] reported rates of NICU admission for IVF and IUI, but lacked a *p*-value for the between-group comparison. Poon et al. [28] reported higher rates for NICU admission following IVF compared to NC, while differences were not significant when comparing IUI and NC (Table 2).

### 5.4. Other

In addition to these results, the APGAR score [25,26,28] and perinatal mortality [26,27,28] were included to a certain degree within three studies. None of the studies found differences in these outcomes between IVF and IUI. Small for gestational age [27] and birth defects [28] were reported by one study each and were not found to differ significantly between groups. More specific neonatal complications, such as respiratory distress, were reported in some studies [26,28], suggesting no significant differences between IVF and IUI conceptions.

### 5.5. Summary

To conclude, neonatal outcomes were reported in a more uniform manner. Studies’ results for some of the outcomes vary; however, current evidence does not suggest large differences in neonatal outcomes between IVF and IUI. Their results should be carefully interpreted, considering each study’s strengths and limitations (Table 3).

## 6. Context from ART vs. Non-ART Comparisons

Large cohort studies and recent narrative/systematic reviews consistently report higher risks of preterm birth, lower birthweight, and select adverse child health outcomes among ART-conceived offspring compared with spontaneously conceived peers [16,17]. These findings provide an external benchmark suggesting that, if laboratory and stimulation exposures contribute to risk, gradients might exist across the spectrum of spontaneous conceptions, IUI, and IVF. Our synthesis was consistent with this hypothesis, but the effect estimates are imprecise, and heterogeneity is substantial. Consequently, IUI–IVF differences should be contextualized against non-ART comparators to avoid over- or under-attribution of risk to treatment modality alone. This further highlights a major limitation of the existing IVF–IUI literature: the majority of comparative studies predate these contemporary ART advancements, which may attenuate or obscure differences between treatments.

In addition, high-quality population-based studies provide strong insight into whether adverse perinatal outcomes arise from infertility itself or from treatment-related factors. Large cohorts consistently demonstrate that ART-conceived pregnancies have higher risks of preterm birth, low birthweight, and hypertensive disorders compared with spontaneous conceptions, even after adjusting for parental characteristics and infertility diagnoses [5,12,13,32,33]. These findings support the presence of treatment-related contributors, including ovarian stimulation, embryo culture environment, and endometrial preparation, which are known to differ significantly across ART modalities [34,35]. Collectively, this pattern supports a graded risk model from spontaneous conception→IUI→IVF, underscoring the value of IVF–IUI comparisons for separating treatment-related effects from underlying infertility-related risk.

## 7. Discussion

The existing literature comparing ART and spontaneously conceived pregnancies consistently demonstrates higher risks of preterm birth, low birthweight, hypertensive disorders, and other adverse neonatal outcomes among ART-conceived offspring [12,13,33]. Importantly, several population-based studies have shown that these associations persist even after accounting for maternal age, infertility diagnoses, and baseline health factors [32,35], suggesting that aspects of the treatment process—rather than infertility alone—likely contribute to these risks. This broader context is essential when interpreting the comparatively small or inconsistent differences observed between IUI and IVF in the present review. If treatment-related exposures, such as stimulation intensity, laboratory manipulation, or endometrial preparation, influence perinatal outcomes, then IVF—characterized by a greater hormonal exposure and embryo handling than IUI—would logically fall further along a graded risk continuum extending from spontaneous conception to IUI and then to IVF.

The patterns observed in our synthesis broadly align with this concept of a dose–response relationship, although estimates remain imprecise due to small sample sizes and methodological heterogeneity across included studies. Mechanistic work also supports this gradient, as differences in ovarian stimulation, embryo culture, and endometrial programming have each been associated with variations in obstetric and neonatal risk [34,35]. However, because most IVF–IUI comparative studies were conducted before the implementation of modern ART innovations such as vitrification, single-blastocyst transfer, and contemporary stimulation protocols, true differences may be attenuated or obscured. Situating the IVF–IUI comparison within the broader ART vs. non-ART literature, therefore, strengthens the interpretation of our findings and underscores the need for updated, well-controlled studies reflecting current clinical practice.

## 8. Limitations

We restricted our study to singletons and excluded donor sperm and twin conceptions from pooled interpretation to minimize confounding from multifetal gestation and immunologic exposure.

There are significant limitations present in almost all studies (Table 3). A major overarching limitation is the age of the evidence: four of the included studies were performed before 2010 [25,26,30,31], and several nearly 20 years ago [25,26,30]. Because ART has evolved substantially—most notably with vitrification, improved embryo culture systems, single-embryo transfer, milder stimulation protocols, and enhanced luteal support—the outcomes reported in these older cohorts may not reflect current practice. As a result, any true differences between IVF and IUI may be underestimated or obscured by outdated methodologies and technology. Another major limitation was the limited sample sizes, with the majority of studies including fewer than 600 pregnancies distributed between both groups [25,26,28,31]. Moreover, in three studies [26,28,31], there were large differences in group sizes. Sagot et al. [31] only evaluated for congenital defects between the IVF and IUI comparison groups. Two of the studies had no information about the infertility diagnoses and the stimulation protocol used [28,31], while De Sutter et al. [25] and Wessel et al. [29] reported different stimulation used in the IVF and IUI groups. None of the studies reported any cycle characteristics, such as peak estradiol and endometrial thickness. These limitations impeded the implementation and generalization of the results. More well-designed and high-quality studies that address these limitations are required to determine the impact of infertility and fertility treatments on maternal and neonatal outcomes.

A further limitation is representativeness. Several included studies enrolled comparatively few IUI pregnancies or reported IUI arms that constituted a small fraction of the total cohort, reducing precision and potentially biasing comparisons. Moreover, much of the underlying evidence predates contemporary practice (e.g., single-blastocyst transfer, vitrification, milder stimulation, improved culture systems), limiting external validity to today’s patients. Finally, most studies lacked a concurrent non-ART comparator, which impedes separation of infertility-related risk from treatment-related risk—an approach supported by recent reviews advocating explicit ART vs. non-ART contrasts [16,17]. Taken together, the reliance on older datasets substantially limits generalizability and highlights the need for contemporary, adequately powered studies using standardized reporting and modern ART protocols.

## 9. Conclusions

It is well established that pregnancies following ART are at increased risk for maternal and neonatal complications. There is a growing interest in the literature to differentiate whether the increased maternal and neonatal complications observed in pregnancies conceived via fertility treatments are attributed to the diagnosis of infertility or the fertility treatment used. Taken together, current evidence is insufficient to draw firm conclusions about differential maternal and neonatal risks between IVF and IUI because almost all studies have significant limitations that hinder clear interpretation, including sample size limitations and protocol heterogeneity. This uncertainty is driven in large part by the age of much of the available data, which predates modern IVF and IUI techniques, and therefore may not capture clinically meaningful differences that exist today. Future research should include adequately powered, contemporary cohorts with standardized exposure and outcome reporting, incorporate a non-ART comparator arm, and—where possible—report process-level modifiers—for IUI (agent class, follicle number, peak estradiol, trigger use) and for IVF (fresh vs. frozen transfer, culture duration, ICSI, endometrial protocol)—to move beyond modality labels toward actionable, mechanism-informed risk mitigation.

## Figures and Tables

**Table 1:** Study level estimates on maternal outcomes.

Outcome	IVF vs. IUI Comparison
Mode of delivery	De Sutter et al. [25]: cesarean section rates: 21.0% for IVF vs. 27.8% for IUI, *p* = 0.256 Nuojua-Huttunen et al. [26]: cesarean section rates: 25.4% for IVF vs. 25.0% for IUI, *p*-value not reported Malchau et al. [27]: cesarean section rates: 27.6% for IVF vs. 27.7% for IUI, *p* = 0.925 Poon et al. [28]: OR (95%CI) for c-section: IVF vs. NC: 2.401 (1.878–3.069); IUI vs. NC: 0.947 (0.460–1.952)
Antepartum hemorrhage	De Sutter et al. [25]: first trimester: 16.3% for IVF vs. 21.4% for IUI, *p* = 0.337; second trimester: 6.5% for IVF vs. 5.6% for IUI, *p* = 1.000; third trimester: 5.7% for IVF vs. 5.0% for IUI, *p* = 1.000 Poon et al. [28]: OR (95%CI) for antepartum hemorrhage: IVF vs. NC: 3.309 (2.026–5.403); IUI vs. NC: 2.482 (0.595–10.348) Wang et al. [30]: 8% for IVF vs. 3.2% for IUI, *p* = 0.001
Hypertensive disorders of pregnancy	De Sutter et al. [25]: 15.1% for IVF vs. 9.5% for IUI, *p* = 0.263 Nuojua-Huttunen et al. [26]: eclampsia risk: 0.4% for IVF vs. 1.1% for IUI, *p*-value not reported Malchau et al. [27]: 5.6% for IUI vs. 4.8% for IVF, *p* = 0.110 Poon et al. [28]: OR (95%CI) for preeclampsia: IVF vs. NC: 1.679 (1.068–2.637); IUI vs. NC: 0.518 (0.071–3.784) Wessel et al. [29]: 2.2% for IVF vs. 6.2% for IUI, *p*-value not reported

Abbreviations: odds ratio (OR), in vitro fertilization (IVF), natural conception (NC), intrauterine insemination (IUI), 95% confidence interval (95%CI).

**Table 2:** Study level estimates on neonatal outcomes.

Outcome	IVF vs. IUI Comparison
Gestational age (GA) at delivery	De Sutter et al. [25]: GA (days): 272.8 ± 25.0 for IVF vs. 271.9 ± 15.5 for IUI, *p* = 0.402 Nuojua-Huttunen et al. [26]: GA (weeks): 39.4 ± 2.2 for IVF vs. 39.5 ± 1.8 for IUI, *p*-value not reported Malchau et al. [27]: GA (days): 275.8 ± 15.9 for IVF vs. 277.5 ± 13.2, *p* < 0.001 Poon et al. [28]: OR for PTB: IVF vs. NC: 1.571 (0.955–2.585); IUI vs. NC: 1.253 (0.301–5.215); VPTB: no cases for either IVF or IUI Wang et al. [30]: OR for PTB: IVF vs. NC: 2.39 (1.71–3.34); IUI vs. NC: 1.50 (1.01–2.02) Wessel et al. [29]: GA (weeks): 39.3 ± 1.4 for IVF vs. 39.2 ± 2.2 for IUI, *p*-value not reported Sagot et al. [31]: GA (weeks): 38.5 ± 2.6 for IVF vs. 38.7 ± 2.5 for IUI, *p*-value not reported
Birth weight (BW)	De Sutter et al. [25]: BW (grams): 3140 ± 633 for IVF vs. 3157 ± 670 for IUI, *p* = 0.552 Nuojua-Huttunen et al. [26]: BW (grams): 3363 ± 611 for IVF vs. 3285 ± 575 for IUI, *p*-value not reported Malchau et al. [27]: BW (grams): 3356 ± 600 for IVF vs. 3434 ± 571 for IUI, *p* < 0.001 Poon et al. [28]: OR for LBW: IVF vs. NC: 1.161 (0.807–1.671); IUI vs. NC: 0.665 (0.204–2.163); VLBW: IVF vs. NC: 1.736 (0.942–3.202); no cases for IUI; ELBW: IVF vs. NC: 2.119 (0.861–5.211); no cases for IUI Wessel et al. [29]: BW (grams): 3467 ± 436 for IVF vs. 3291 ± 558 for IUI, *p*-value not reported
NICU admission	De Sutter et al. [25]: 12.8% for IVF vs. 19.4% for IUI, *p* = 0.151 Nuojua-Huttunen et al. [26]: 4.4% for IVF vs. 2.2% for IUI, *p*-value not reported Malchau et al. [27]: 12.1% for IVF vs. 10.5% for IUI, *p* = 0.080 Poon et al. [28]: OR for NICU admission: IVF vs. NC: 1.625 (1.078–2.449); IUI vs. NC: 1.728 (0.612–4.879)
APGAR score	De Sutter et al. [25]: % of children with APGAR < 7 after 1 min: 7.1% for IVF vs. 6.7% for IUI, *p* = 0.563; after 5 min: 1.8% for IVF vs. 1.0% for IUI, *p* = 0.574 Nuojua-Huttunen et al. [26]: % of children with APGAR < 7: 6.9% for IVF vs. 6.5% for IUI, *p*-value not reported Poon et al. [28]: OR for APGAR ≤ 5 at 5 min: IVF vs. NC: 2.315 (0.561–9.552); no cases after IUI
Perinatal mortality	Nuojua-Huttunen et al. [26]:7.2% for IVF vs. 10.9% for IUI, *p*-value not reported Malchau et al. [27]: 1.5% for IVF vs. 1.1% for IUI, *p* = 0.100 Poon et al. [28]: no cases after either IVF or IUI

Abbreviations: preterm birth (PTB) (<37 weeks), odds ratio (OR), in vitro fertilization (IVF), natural conception (NC), intrauterine insemination (IUI), 95% confidence interval (95%CI), very preterm birth (VPTB) (<32 weeks), low birth weight (LBW) (<2500 g), very low birth weight (VLBW) (<1500 g), extremely low birth weight (<1000 g), neonatal intensive care unit (NICU. Adjusted for age, race/ethnicity, level of education, nulliparity, body mass index, and gestational age at time of delivery.

**Table 3:** Studies’ limitations.

Study (Ref.)	Sample Size and Group Balance	Era	Exposure and Cycle-Level Data	Confounding Control and Baseline Data	Notes (Representativeness/Scope)
De Sutter et al. (2005) [25]	n = 252 (126 IUI/126 IVF); questionnaire-based (recall bias)	>15 years old	Different stimulation across groups: IUI Natural/CC vs. IVF gonadotropins; no cycle-level characteristics	Limited baseline covariates; no lifestyle data; no stats for rare events	Treatment sequence bias (mild cases often start with IUI)
Nuojua-Huttunen et al. (1999) [26]	n = 444 total; IUI = 111, IVF = 333; 14 IUI missing data	>20 years old	No cycle-level characteristics reported	Group imbalance; high multiples complicate matching; SES/residence differences	Small IUI arm limits precision
Malchau et al. (2014) [27]	IUI singletons n = 6338 (registry); IVF comparator reported separately	~10 years old	Few baseline covariates (age, nulliparity, BMI, smoking only); no cycle-level characteristics	Infertility causes recorded but not specified	Time-to-birth available for only ~1/6
Poon et al. (2013) [28]	ART n = 589; IUI = 39, OI-CC = 11, IVF/ICSI = 536 (highly imbalanced)	~10+ years old	No baseline characteristics; no stimulation/diagnosis details; no cycle-level characteristics	Large group imbalance; limited covariate control	Indirect comparisons via NC; very small IUI arm
Wang et al. (2002) [30]	Size not reported per arm here; includes NC comparator	≈20 years old	No cycle-level characteristics reported	NC lacks prior PTB/SAB, race, SES, smoking; groups differ by age, infertility length, smoking, parity, threatened miscarriage, abruption	Residual confounding likely
Wessel et al. (2021) [29]	n = 472 livebirths; unexplained/mild male factor only	Contemporary	Different stimulation: IUI gonadotropin + CC; IVF gonadotropin + natural; no cycle-level characteristics	No patient characteristics reported; no direct IUI vs. IVF (only indirect vs. NC)	Restricted external validity due to inclusion criteria
Sagot et al. (2012) [31]	IVF = 1265 vs. IUI = 635 (≈2:1 imbalance); 18 centers (France)	>10 years old	No inclusion diagnoses; no stimulation details; no cycle-level characteristics	Voluntary data submission; inter-center variability	Only congenital anomalies compared; terminations < 22 weeks not recorded

Abbreviations: intrauterine insemination (IUI), natural conception (NC), in vitro fertilization (IVF), assisted reproductive technology (ART), clomiphene citrate (CC), body mass index (BMI), preterm birth (PTB), spontaneous abortion (SAB), socio-economic status (SES).

## Data Availability

Not applicable.
